# HPV knowledge and vaccine acceptability: a survey-based study among parents of adolescents (KAPPAS study)

**DOI:** 10.1186/s13027-022-00467-7

**Published:** 2022-11-17

**Authors:** Noelia López, Ignacio Salamanca de la Cueva, Elena Taborga, Auxiliadora Fernández de Alba, Inés Cabeza, Reyes Mazas Raba, Josep Marès, Patricia Company, Bruno Herrera, Manuel Cotarelo

**Affiliations:** 1 Medical Affairs Department, MSD Spain, C/Josefa Valcarcel, 38, 28027 Madrid, Spain; 2 Instituto Hispalense de Pediatría, Seville, Spain; 3 Centro de Salud de Villalegre - La Luz, Avilés, Asturias Spain; 4 Healthcare Centre Galdakao, Vizcaya, Spain; 5 Healthcare Centre Gama, Cantabria, Spain; 6 Institut Pediàtric MARES-RIERA, Girona, Spain; 7 Healthcare Centre Pla, Elche, Alicante Spain

**Keywords:** HPV, HPV vaccine, Vaccination, Knowledge, Acceptability, HPV-related diseases, Adolescent, Parents

## Abstract

**Background:**

Human papillomavirus (HPV) infection is recognized as one of the major causes of infection-related cancer worldwide. In Spain, the HPV vaccination program started in 2007 and until 2022, it targeted 12-year-old girls.

**Methods:**

This was a cross-sectional, multicenter survey-based research carried out at 24 pediatric offices to describe HPV knowledge and vaccine acceptability in parents of children aged between 9 and 14 years-old in Spain. Parents were randomly selected from the medical records following specific quotas to ensure representativeness. The survey included five sections that aim to collect information about sociodemographic characteristics, knowledge of HPV, knowledge and acceptability of vaccines in general, HPV vaccination knowledge and HPV vaccine acceptability. Each section was constituted by a number of close questions with different answer options. Specific scores were assigned to each possible answer to these questions. Based on these scores, four composite variables were created to assess HPV knowledge, HPV vaccine knowledge, HPV vaccine acceptability and vaccines knowledge and acceptability in general. A latent class analysis was performed to identify different group of respondents according to their HPV vaccine acceptability.

**Results:**

A total of 1405 valid surveys were included, with 86.19% of the respondents being mothers. The mean score of HPV knowledge was 28.92 out of 40 (maximum value) (95% CI 28.70–29.20) and the mean score of HPV vaccine acceptability was 3.37 out of 5 (maximum value). One third of parents still need more information to take a final decision about HPV vaccination in their children. Parents perceived that females were more likely to become infected than males and tended to associate HPV infection mainly with cervical cancer, showing a. a lack of information about other HPV-related diseases affecting males.

**Conclusions:**

This study results highlight the need for future actions and educational initiatives to raise awareness of HPV consequences in both genders and to contribute to achieving the elimination of HPV-related diseases beyond cervical cancer.

**Supplementary Information:**

The online version contains supplementary material available at 10.1186/s13027-022-00467-7.

## Background

Human papillomavirus (HPV) infection is recognized as one of the major causes of infection-related cancer worldwide, as well as a causal factor of other diseases such as genital warts or recurrent respiratory papillomatosis [1].

Nowadays, HPV infections are regarded as the most common sexually transmitted infections in the world. Indeed, 80% of sexually active people will become infected at some point in their lifetime. However, most of these infections are typically controlled immunologically within 1–2 years, although if they persist, they can cause different types of cancer, such as cervical cancer or oropharyngeal cancer [1–3]. Although the HPV-related burden of disease used to be higher in females than in males in most countries, the latest epidemiological studies point to an increasing trend in the incidence of anal and oropharyngeal cancer in men [4]. Immunization against HPV infection is the most promising strategy for the prevention of one of the most common sexually transmitted infections worldwide [1].

In 2020, the World Health Organization/The United Nations Children's Emergency Fund (WHO/UNICEF) published the Estimates of National HPV Immunization Coverage from 2010 to 2019. According to this publication, 107 (55%) of the 194 WHO Member States had introduced HPV vaccination [5]. America and Europe are by far the WHO regions with the most introductions, and 85% and 77% of their countries, respectively, have already introduced the HPV vaccination, with almost one third of the programs (33 out of 107) being gender-neutral [5]. Many countries have recognized the value of HPV gender-neutral vaccination programs for the purpose of achieving the goal of eliminating not only cervical cancer but all HPV-related diseases [6]. According to the latest data published by the European Cancer Organization in 2020, 26 countries in the European region of the WHO are or will be including boys in their national HPV vaccination programs [6]. This represents almost half (48%) of all the countries in this region [6]. In Spain, HPV vaccination was introduced into the national immunization program in 2007–2008. Until 2022, it targeted 12-year-old girls [7], with a mean vaccination coverage rate (VCR) of 81.8% in 2020 (latest data available) [8].

The latest vaccination calendar published by the Spanish Association of Pediatricians (AEP), dated January 2022, recommends HPV vaccination for girls and boys at the age of 12 years [9]. However, although this Society [9] recommends including boys in HPV vaccination programs since 2018, the funded Spanish Immunization Program continues to target only female adolescents and specific high-risk groups. Regardless of gender, it is important to stress that the parents of adolescents play a key role in vaccination decision-making, and the success of the vaccination program relies largely on parental decision-making [10].

Our group published a systematic literature review in 2020 about HPV knowledge and vaccine acceptability among European adolescents and their parents. This review concluded that since HPV knowledge and vaccine acceptability were still modest and varied widely between studies across EU countries, coordinated efforts should be made to provide the relevant population with information to allow informed decision-making on HPV vaccination [10]. If the information received by the parents is not properly balanced there might be a negative impact on HPV vaccine acceptability and therefore the HPV-related disease elimination goal would not be achievable [6].

To our knowledge, as yet no studies to assess knowledge of HPV and vaccine acceptability among parents of children (girls and boys) have been performed at national level in Spain. Spanish studies already published are mainly regional and have focused exclusively on describing acceptability in the female or adult populations at regional level [11–13]. As the VCR differs substantially between regions, a national study to evaluate knowledge of HPV and vaccine acceptability for girls and boys is called for.

The objective of this study, KAPPAS study (Knowledge and Acceptability of Papillomavirus Vaccines in Parents of Adolescents in Spain*),* was to describe HPV knowledge and HPV vaccination acceptability among parents of girls and boys aged between 9 and 14 years living in Spain. Moreover, the study assessed the correlation between HPV knowledge and HPV vaccine acceptability and the influence of different sociodemographic variables.

This paper focuses on the results of the level of HPV knowledge and vaccine acceptability in parents of adolescents in Spain. The analysis of the factors involved in HPV knowledge and vaccine acceptability has been addressed in a separate publication [14].

## Methods

### Study design and setting

This was a cross-sectional, multicenter survey-based research carried out at twenty-four (public and private) pediatric offices in Spain between May 2019 and April 2020. Sample size and sample distribution was estimated to assess primary and secondary endpoints with appropriate precision (≤ 5%) per each stratum of interest.

Due to the COVID-19 pandemic, only surveys completed before 16 April were considered valid for the analysis to ensure external validity of our results; as COVID-19 pandemic could interfere in vaccine perceptions in the overall population. The study recruited the fathers, mothers or legal guardians of children (girls and/or boys) aged between 9 and 14 years who had been living in Spain for at least the last 12 months.

The study obtained the favorable approval of the reference Investigational Ethical Committee (IEC). The rest of IECs requested the evaluation or only the registration of the protocol as necessary per their local guidance.

### Survey development

A structured survey, in Spanish language, was developed to collect epidemiological variables as well as knowledge- and acceptance-related measurements. It was designed based on a previous systematic literature review [11] carried out by our group to identify published studies and items used to evaluate parental and/or adolescent HPV knowledge and/or HPV vaccination acceptability. Based on the inputs found in the systematic review, a draft questionnaire was developed and then validated by an Expert Committee comprised of 4 pediatricians who were experts in adolescents and HPV. The draft questionnaire was afterwards tested through cognitive debriefing methodology on a representative sample of 12 parents of children between 9 and 14 years old following a fine-tuning of the wording and comprehensiveness of the questionnaire according to participants’ perceptions and suggestions. The Experts Committee validated the final version of the questionnaire.

The final survey (Additional file 1: Material S1) included five sections: (1) *sociodemographic characteristics* (15 items); (2) *knowledge of HPV* (9 items); (3) *knowledge of vaccines and their acceptability in general* (5 items); (4) *HPV vaccination knowledge* (8 items); (5) *HPV vaccine acceptability* (7 items). All questions were closed with multiple choice of answers, using the appropriate scale of response according to the specific type of question (yes/no, yes/no/not sure, ordinal scale of level of agreement or specific response options, when needed) (Additional file 1: Material S2).

### Data collection

Representative centers across different Spanish regions were invited to participate. A preliminary selection of sites was performed by the Committee of Experts of the study-a stratification based on HPV VCR was performed to ensure adequate representativeness. To those centers preliminary selected, a feasibility questionnaire was sent, to ensure that their willingness and availability to participate in this study, and to ensure that they had enough patients between 9 and 14 y.o in order to be able to send the questionnaire to the parents of those patients.. Active recall recruitment process was designed to prevent any selection bias such as chronically ill patients who might attend pediatricians’ offices more frequently (Additional file 1: Figure S1). All children aged between 9 and 14 years were identified from investigator databases or medical records and were divided into 4 stratification quotas based on gender and age (males 9–11 y.o; females 9–11 y.o,; males 12–14 y.o and females 12–14 y.o). Spanish regions were classified into 2 categories according to their HPV VCR: low VCR: < 77.8% / high VCR: ≥ 77.8%). In 2107, when the protocol was drafted, the mean national VCR was 77.8%.

The investigators actively invited parents following the order generated by a randomization tool by telephone and according to the stratification quotas to ensure representativeness. The survey could be completed either online or in a paper format in an autonomous manner. The investigator sent the participant an e-mail/mobile text message with a link to the survey and a code. Each participant could access the link, introduce the code and confirm his/her acceptance to participate and afterwards fill the online survey (Additional file 1: Figure S1). For the paper-pen option, the participant could either collect the questionnaire in the office or print it directly from the same link. After completion, the questionnaire could be sent through pre-paid envelopes or be delivered at the doctor’s office. Per protocol, the survey should not be completed in the presence of the pediatrician, nor should anyone from his team interfere with the participant’s answers.

### Statistical analysis

A descriptive analysis of the qualitative and quantitative variables was performed. The qualitative variables were described by means of frequencies and percentages. Normality data test were performed to choose the appropriate statistical tests accordingly. The quantitative variables were described by n, mean, standard deviation, 95% confidence interval (CI), median, interquartile interval (25^th^ and 75^th^ percentiles) and minimum and maximum according to the distribution. The CI was calculated with the Clopper-Pearson method for binomial proportions and the Sison and Glaz (1995) method for multinomial proportions. The Pearson’s correlation coefficient was calculated to study the correlation between quantitative variables. 95% CI were built using a bootstrapping method (N = 1000 iterations).

Four composite variables on knowledge and acceptability were created based on the responses to the items in sections 2–5 of the questionnaire. The points were summated to create a total score and the results were described with the mean and 95% CI. Details on score assignment can be found in Additional file 1: Material S2.

*Degree of HPV knowledge* total score ranged from 0 to 40, *Degree of HPV vaccine acceptability* ranged from 0 to 5, *Degree of HPV vaccination knowledge* ranged from 0 to 21 and *Degree of knowledge of vaccines and their acceptability in general* ranged from minus 10 to 10.

In order to identify the different profiles of parents who answered the survey, a latent class analysis (LCA) was conducted. The different response patterns were used to classify parents into groups (classes) according to answer similarities. LCA uses categorical data to create the groups, and the results provide the probability of a respondent belonging to each class. The questions included in the LCA were selected based on the previous assessment on their relevance in HPV vaccine acceptability: 2.4, 2.5, 3.1–3.5, 4.6, 4.7 and 5.1–5.4.

## Results

A total of 3110 participants were selected and contacted. 1071 did not answer and 555 refused participation, which translates into a response rate of 47.7%. After exclusion of unanswered and the invalid surveys, (n = 79) 1405 surveys were considered valid for the analysis (1116 online and 289 paper-based) (see reasons of invalid surveys in Additional file 1: Figure S2).

### Sociodemographic characteristics

Most of the recruitment sites were public (68.0%) and were located in a region considered as low VCR (55.9%) (Table 1). Distribution according to gender and age was similar in each stratification group.

**Table 1:** Setting and socio-demographics profile of respondents

Characteristics		N (%)
Type of center	Public	956 (68.04)
	Private	449 (31.96)
Vaccine coverage	High	620 (44.13)
	Low	785 (55.87)
Distribution	Girls 9–11 years	372 (26.48)
	Girls 12–14 years	364 (25.91)
	Boys 9–11 years	335 (23.84)
	Boys 12–14 years	334 (23.77)
Relation to the child	Father	194 (13.81)
	Mother	1211 (86.19)
Age, years	≤ 29	63 (4.48)
	30–39	218 (15.16)
	40–49	971 (69.11)
	50–59	152 (10.82)
	≥ 60	1 (0.00)
Educational level	No formal schooling	4 (0.00)
	Elementary	170 (12.10)
	Secondary	152 (10.82)
	High school	145 (10.32)
	Vocational training	319 (22.70)
	College	500 (35.59)
	Master	115 (8.19)
Work status	Student	15 (1.07)
	Full-time worker	868 (61.82)
	Half-time worker	253 (18.02)
	Unemployed	240 (17.09)
	Retired	20 (1.42)
	Temporary disabled	8 (0.60)
Place of residence	Less than 2000 inhabitants	108 (7.69)
	2000–10,000 inhabitants	294 (20.94)
	10,000–50,000 inhabitants	482 (34.33)
	More than 50,000 inhabitants	520 (37.04)
Nationality	Spanish	1368 (97.37)
	Other	37 (2.63)
Marital status	Single	75 (5.34)
	Married	1148 (81.71)
	Divorced	169 (12.03)
	Widower	13 (0.93)
Own vaccination status	No	1076 (76.58)
	Yes	107 (7.62)
	Not sure	222 (15.80)
Number of children	1	294 (20.93)
	2	886 (63.06)
	3	193 (13.74)
	4 or more	32 (2.28)

Most respondents were mothers (86.2%), between 40 and 49 years (69.1%), with a university degree (35.6%), in full-time employment (61.8%), living in a place with more than 50,000 inhabitants (37.0%), of Spanish nationality (97.4%), married (81.7%), with 2 children (63.1%) and not vaccinated against HPV (76.6%). Only 7.6% of the parents stated that they had been vaccinated against HPV, and 15.8% of them were unsure of their vaccination status (Table 1). Of the children about whom the survey was completed, 736 (52.4%) were girls with a mean age of 11.5 (SD: 1.6) years. Eight hundred and ninety-five (63.7%) of the children had not been vaccinated against HPV and 391 (27.8%) had. For the rest, the vaccination status was reported as “unknown”.

### HPV knowledge

The majority of the respondents (90.7%) had heard of HPV infection. The pediatrician (44.8%) was the most common source of information, followed by family and friends (40.4%) and the Internet (39.3%).

The participants had a medium-to-high degree of HPV knowledge, with a mean score of 28.9 out of 40 (95% CI 28.7–29.2). Additional data are provided in the Additional file 1: Figure S3 and S4. In general, the parents agreed that HPV was a serious health problem (39.9% strongly agree, 53.1% agree) and that it was one of the most common sexually transmitted diseases (17.9% strongly agree, 52.2% agree).

**Fig. 1:**
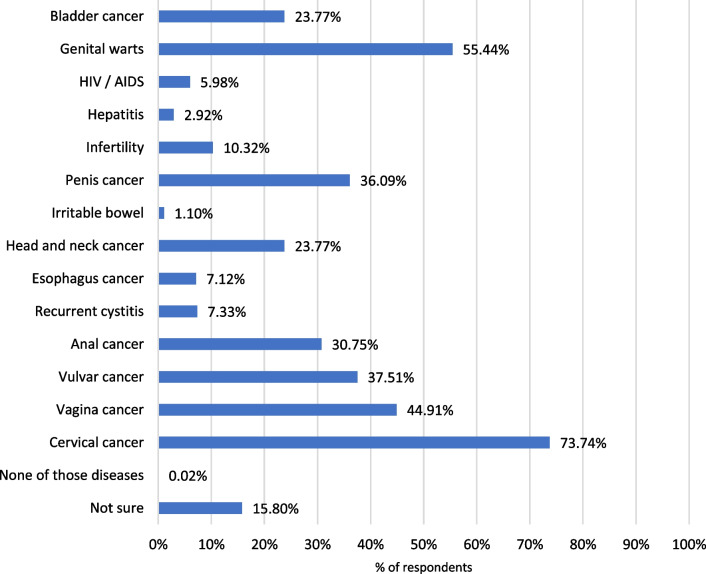
Diseases related to HPV

The respondents correctly answered that HPV is a sexually transmitted disease (89.2%), although 10.8% were not sure how it is transmitted (Additional file 1: Figure S5). Most of the parents considered than women (89.2%) or girls (69.1%) could get infected by HPV. In contrast, only 50.2% and 67.2% considered that boys and men could be infected, respectively (data not shown). Regarding possible diseases related to HPV infection, the majority of the respondents considered HPV infection to be related to cervical cancer (73.7%). However, in Fig. 1 it is shown that parents were less aware of the role of HPV in other diseases.

In terms of HPV prevention; most of the parents answered that it could be prevented by the HPV vaccine (87.2%) and condom use (80.9%). A lower proportion stated that delayed sexual debut (10.2%), personal hygiene (13.9%) and monogamy (14.2%) were also reported mechanisms for preventing HPV infections. Seven percent were not sure how to prevent this infection (data not shown). Additional data are provided in the Additional file 1: Figure S5.

In order to obtain more information about HPV infection, most parents would rather consult healthcare professionals such as pediatricians (80.1%), family doctors (68.3%) and gynecologists (78.1%). Only 19.8% of the respondents would consult the Internet or the social media (Data not shown).

### HPV vaccine acceptability

The respondents had a medium-to-high degree of HPV vaccine acceptability, translating into a mean score of 3.37 out of 5 (95% CI 3.30–3.44) (Additional file 1: Figure S3).

**Fig. 2:**
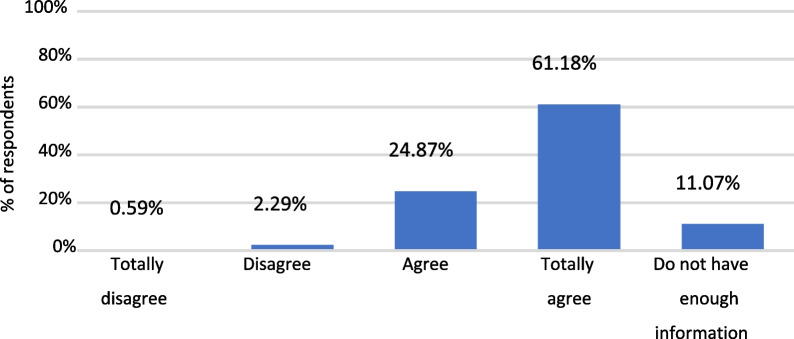
Answers to question: “I would vaccinate my son/daugther against HPV”

In general, the participants presented a high level of agreement (strongly agree + agree) in considering that HPV vaccination is necessary in girls and boys. However, the results revealed that a higher proportion of parents considered it necessary in girls compared to boys (Fig. 2 and Additional file 1: S8).

The main reasons for having the child vaccinated were to protect them against sexually transmitted diseases (67.4%) or against cancer and/or genital warts (77.4%), whereas the reasons for not having them vaccinated included lack of information (27.9%), fear of possible adverse events (20.9%) and other unspecified reasons (29.3%) (Additional file 1: Figure S7).

With regard to the type of information needed for the HPV vaccination to be acceptable for parents who initially disagreed to vaccinate their daughter/son: 55.7% would request information about vaccine safety; 54.5% would need a doctor’s recommendation, 51.8% about HPV vaccine efficacy, 49% about the HPV vaccine in general and 46.1% about HPV infection (Data not shown).

**Fig. 3:**
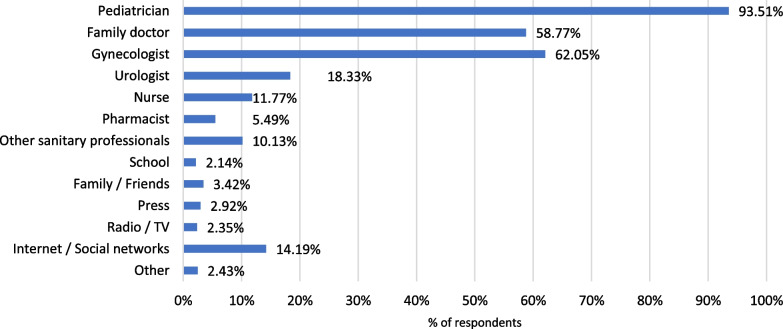
Main sources to be consulted for obtaining more information about HPV vaccine, according to participant’s opinion

The proportion of parents that would consult a pediatrician to obtain further information about the HPV vaccine represented 93.6% (Fig. 3).

### Knowledge of HPV vaccination

The parents evinced an intermediate-to-high degree of knowledge of HPV vaccination, with a mean score of 15.5 out of 21 (95% CI 15.3–15.6) (Additional file 1: Figure S3 and S4).

As it was observed with HPV infection, 92.1% of the respondents had heard of the HPV vaccine, the main source of information being the pediatrician (62.3%). Family and friends (34.5%), the gynecologist (27.8%) and the Internet (25.1%) were also mentioned as sources of information about the HPV vaccination.

Only 57.5% of the participants provided a correct answer indicating that the HPV vaccine was funded only for girls, whereas up to 25.1% of the parents did not know if the HPV vaccine was funded as part of the Spanish vaccination program (data not shown).

When the parents were questioned about the recommended age for vaccination, most of them answered correctly, with a mean (SD) reported age of 12.10 (1.21) years.

As occurred with HPV infection, 90.8% and 55.2% of the parents knew that girls and women, respectively, can be vaccinated against HPV. In contrast, the proportion of participants that considered that male populations could be eligible for the HPV vaccine was lower: only 60.1% and 37.9% of the parents considered that boys and men, respectively, could be vaccinated (Data not shown).

Up to 75.9% of the parents concurred (strongly agree + agree) in considering the HPV vaccine as effective, and 76.8% agreed that its benefits outweigh the risks. Nevertheless, it is important to point out that nearly 20% of the parents opined that they lacked sufficient information to answer (data not shown).

**Fig. 4:**
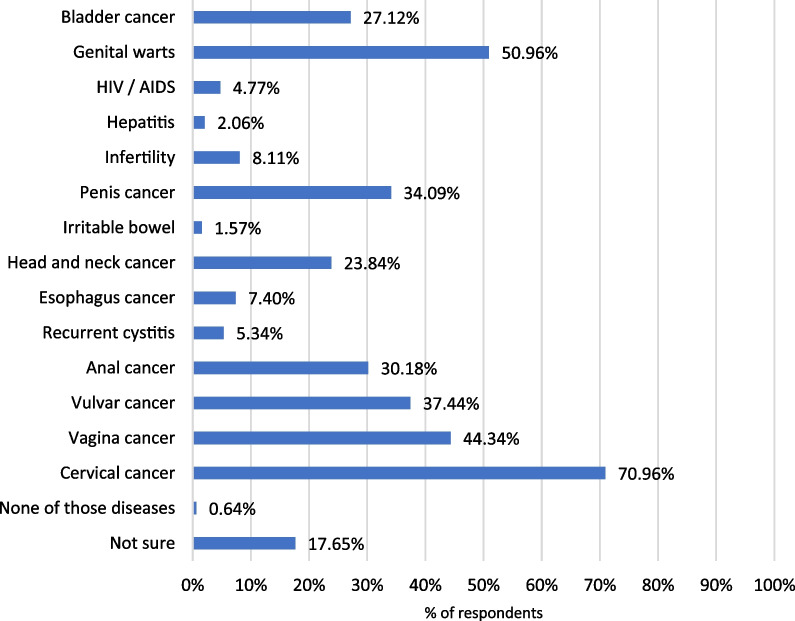
Diseases that can be prevented by HPV vaccination, according to participant’s opinion

The HPV vaccine results tallied with the answers in the HPV knowledge section, HPV-related diseases. Thus, 80.0% of the participants considered that cervical cancer could be prevented with the vaccine, although the percentage was much lower for other diseases, such as genitals warts or anal cancer, that also affect males (Fig. 4). Additional data are shown in Additional file 1: Figure S6.

### Knowledge and acceptability of vaccines in general

Knowledge and acceptability of vaccines was high, 6.6 out of 10 (95% CI 6.4–6.8). In fact, 25.8% of the participants obtained the maximum score (10 points).

More details are included in Additional file 1: Figure S9.

### Correlations

**Fig. 5:**
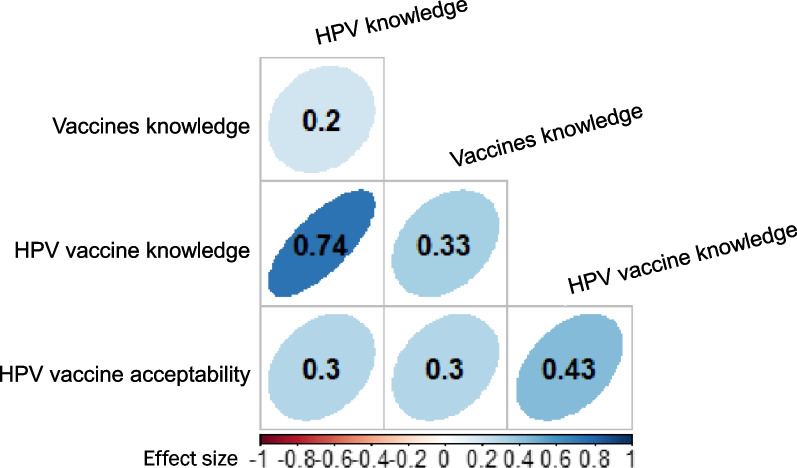
Correlations between knowledge and vaccine acceptability. *p* < 0.001 for all correlations. The colour intensity and shape indicate the strength of the correlation

There were significant and positive correlations between all variables (vaccine knowledge, HPV vaccine knowledge and HPV vaccine acceptability), and parents who scored high in one variable tended to score high in the other variables (Fig. 5).

The highest correlation was observed between HPV knowledge and HPV vaccine knowledge (0.7), followed by the correlation between HPV vaccination knowledge and HPV vaccine acceptability (0.4). Lower correlations were observed between other pairs of variables, although they were statistically significant (Fig. 5).

### Typologies of respondents

The results of the LCA analysis allowed us to conclude that there were 4 groups of parents, according to different response patterns:

- *Class 1* (the probability of belonging to this group was 0.47): they consider that vaccines are useful, effective, beneficial and also that parents who do not have their children vaccinated put other people at risk. On the other hand, they think that both girls and boys should be vaccinated and that their doctor has recommended such vaccination. This group of parents showed a high agreement in considering HPV as a sexually transmitted disease and a serious health problem.
- *Class 2* (probability: 0.34): they consider that vaccines may be useful, effective, beneficial and also that parents who do not have their children vaccinated put other people at risk but that they may need more information. They think that it might be necessary to have both girls and boys vaccinated and that their doctor has recommended such vaccination. This group of parents agreed in considering HPV as a sexually transmitted disease and a serious health problem. Although these parents may have their sons vaccinated, they may need further information about HPV or its vaccine.
- *Class 3* (probability: 0.15): this group of parents are very unsure or lack sufficient information about HPV and its vaccine. While they are not afraid of having their child vaccinated, this lack of information could increase their indecision.
- *Class 4* (probability: 0.04): this group of parents have a higher probability of not accepting the vaccine as they believed that vaccines are not useful, are not safe and are ineffective. In addition, they did not regard HPV as a common sexually transmitted disease and a serious health problem. Furthermore, they tended to think that there is no need to have boys and girls vaccinated. In this group of parents, a high proportion stated that their doctor did not recommend the HPV vaccine.

## Discussion

According to our results, thirteen years after the beginning of the HPV vaccination program in Spain, the degree of knowledge of HPV among parents of adolescents is still modest, although HPV vaccine acceptability is medium–high. There was still a clear tendency to relate HPV to girls and females [6].

Parents perceived that females were more likely to become infected than males and tended to associate HPV infection mainly with cervical cancer. In addition, there was a lack of information about other HPV-related diseases, and even more about those affecting males. These results contrast with the real burden of HPV-related diseases, which is substantial in both genders, causing not only cervical cancer but also anal, penile, vaginal, vulval and oropharyngeal cancers, in addition to genital warts and recurrent respiratory papillomatosis (RRP) [6]. In this context, it is important to highlight that the current objective of HPV vaccination programs in high-income countries is the elimination HPV-related diseases, not only cervical cancer [15]. To this aim establishing gender-neutral vaccination program would therefore seem to be crucial.

Our findings are similar to those of other HPV vaccine awareness studies performed in other European countries, such as the outcomes of our own systematic review published in 2020 [11]. This review found that HPV knowledge and acceptability of the HPV vaccine continued to be modest and varied widely across EU countries, with insufficient information and safety concerns being the main barriers to vaccination acceptability [11]. A more recent review [16] also identified a modest degree of HPV and HPV vaccine knowledge among the male population. In line with the results of previous studies [11, 16–18], the KAPPAS study emphasizes the need to implement actions to provide the relevant population with information, including the possible impact of HPV in males, and therefore empower them to make informed decisions. The significant and positive correlations between HPV knowledge and HPV vaccine knowledge as well as between HPV vaccination knowledge and HPV vaccine acceptability found in our study also underscore the importance of awareness-raising campaigns.

In our study, the LCA revealed that approximately more than one third of the parents may still have insufficient information about HPV and its vaccine and evince a certain indecision in vaccinating their children, particularly boys. This result reinforces the need for educational activities targeting parents that are still hesitant to HPV vaccination to ensure the success of HPV immunization programs. On the other hand, in our study, the percentage of parents who present a higher probability of not accepting the HPV vaccine or vaccines in general is very small (3.80%). This correlated with the high VCR in our country of HPV and the rest of the vaccines, one of the highest in Europe. Our LCA analysis may also help healthcare professionals to identify different types of parents and provide them with balanced information targeting their needs in order to enable them to take informed decisions about their children’s HPV vaccination.

Similar results were published in 2015 in an pan-European study that examined A study published in 2015 examining parental views of HPV vaccination of sons in France, Germany, Italy and the UK [19] found that approximately three quarters of the parents in the UK, Germany and Italy were in favor of the HPV vaccination for their sons. The favorable parents sought to protect their sons from the disease and regarded gender equality as important. Parents in doubt about male HPV vaccination needed more information about HPV diseases and HPV vaccination among males. The rejecting parents were generally skeptical of vaccines and feared the side effects of vaccination. Although all these countries now include boys in their HPV immunization programs, this study was conducted before this inclusion and examined countries with significant differences in vaccination coverage [20, 21]. Parents in countries with high HPV vaccination coverage rates (UK and Italy) tended to recognize the importance of national vaccination programs. Parents in countries with limited HPV vaccination coverage rate (Germany and France) felt a greater need for information from healthcare professionals (HCP) and the public health authorities. The authors concluded that by providing brief information about HPV in both genders, parental acceptance of HPV vaccination for sons could be as high as for girls [19]. This was confirmed by the recent WHO/UNICEF estimations, published in 2020, Bruni et al. [5] which have shown that the VCR in boys and girls is similar in countries with gender-neutral HPV vaccination programs.

Our study is the first of its kind conducted in Spain at a national level that sheds some light on the knowledge and acceptability of HPV and its vaccine.

Some limitations derived from the nature of this study should be borne in mind. Firstly, the potential sources of bias in this study comprised a selection bias due to the parents’ acceptance to participate in the survey and the inclusion of the population entered in medical records. In addition, parent-reported information is subjective and may be affected by social desirability, imprecision or mistakes in interpreting the questions.

## Conclusions

More than 12 years after the implementation of the HPV vaccination program in our country, parental HPV knowledge and vaccine acceptability is medium-to-high. However, HPV is still associated with the female gender, with important lack of knowledge of HPV consequences in males. Moreover, our results also point out that more than one third of parents still need more information to vaccinate their children against HPV. Providing parents with adequate and well-balanced information is crucial to ensure the success of HPV vaccination programs.

## Supplementary Information

**Additional file 1: Material S1.** Questionnaire used in the KAPPAS study. **Material S2**. Scores Keys assigned to each item to compute global scores. **Material S3**. Additional information of methodology and results. **Figure S1**. Recruitment and data collection process. **Figure S2**. Flowchart of participants. **Figure S3**. Total scores and distribution of respondents with regard to A) HPV knowledge, B) HPV vaccine knowledge, C) HPV vaccine acceptability, D) knowledge and acceptability of vaccines in general. **Figure S4**. Box representation of total scores. **Figure S5**. Wrong/Right answers for HPV knowledge. **Figure S6**. Wrong/Right answers for HPV vaccine knowledge. **Figure S7**. Reasons to vaccinated and to not vaccinate the child. **Figure S8**: Responses to questions related to HPV vaccine acceptability. **Figure S9**: Responses to questions related to Knowledge and acceptability of vaccines in general.

## Data Availability

The datasets generated and/or analyzed during the current study are not publicly available due participants privacy protection but are available from the corresponding author on reasonable request.

## Abbreviations

AEP: Spanish Association of Pediatricians
CI: Confidence interval
COVID: Coronavirus disease
EU: European Union
HCP: Healthcare professionals
HPV: Human papillomavirus
IEC: Investigational ethical committee
LCA: Latent class analysis
RRP: Recurrent respiratory papillomatosis
UK: United Kingdom
UNICEF: United Nations International Children's Emergency Fund
VCR: Vaccine coverage rate
WHO: World Health Organization
