# Supplementary material for: HPV knowledge and vaccine acceptability: a survey-based study among parents of adolescents (KAPPAS study)
Source: Infect Agent Cancer. 2022 Nov 17;17:55. doi: 10.1186/s13027-022-00467-7 (PMC9670070; doi:10.1186/s13027-022-00467-7)

**Supplementary Material.**

- **Supplementary Material 1**. Questionnaire used in the KAPPAS study
- **Supplementary Material 2.** : Scores Keys assigned to each item to compute global scores
- **Supplementary Material 3**. Additional information of methodology and results
  - **FIGURE S 1.** Recruitment and data collection process.
  - **FIGURE S 2.** Flowchart of participants.
  - **FIGURE S 3.** Total scores and distribution of respondents with regard to A) HPV knowledge, B) HPV vaccine knowledge, C) HPV vaccine acceptability, D) knowledge and acceptability of vaccines in general.
  - **FIGURE S 4.** Box representation of total scores
  - **FIGURE S 5.** Wrong/Right answers for HPV knowledge
  - **FIGURE S 6**. Wrong/Right answers for HPV vaccine knowledge
  - **FIGURE S 7.** Reasons to vaccinated and to not vaccinate the child.
  - **FIGURE S 8:** Responses to questions related to HPV vaccine acceptability
  - **FIGURE S 9:** Responses to questions related to Knowledge and acceptability of vaccines in general

**Supplementary Material 1. Questionnaire used in the KAPPAS study.**

**Please, remind that the answers provided in this questionnaire should be related to the son/daughter that your healthcare professional has indicated. Please, if possible, try to limit the use of options like “I am not sure/ I don´t know/ I do not have enough information”**

***Sociodemographic characteristics of the respondents.***

| - 1. Please indicate your relationship with the child you are answering for | Father/ legal representative  Mother/ legal representative  Others |
| --- | --- |
| - 1. Please indicate your age | Younger than 29 y.o  30–39 y.o  40–49 y.o  50–59 y.o  60 or older y.o |
| - 1. Please indicate your highest level of education | No formal schooling  Primary education  Lower secondary education  Upper secondary education  Primary and secondary vocational education  Bachelor degree  Master degree/ PhD |
| - 1. Please indicate your current employment status | Student  Full-time employee  Part-time employee  Unemployed  Retired/pensioner  Temporary incapacity |
| - 1. Please indicate the type of your current place of residence | Location of more than 50.000 inhabitants  Location with 10.000 -50.000 inhabitants  Location with 10.000-2.000 inhabitants  Location with less than 2.000 inhabitants |
| - 1. Please indicate your place of birth | Spain  Other country (please select from the list) |
| - 1. Please indicate your nationality | Spanish  Foreigner: please, indicate your country:( select from the list) |
| - 1. In case you don`t have the Spanish nationality, please indicate how long you have been living in Spain | Less than 12 months  12 months or more |
| - 1. Please indicate your civil status | Single  Married or in a stable relationship  Separated or divorced  Widower |
| - 1. How many children (biological/ adopted) do you have? | 1  2  3  4 or more |
| - 1. Please indicate the gender and age of your children | Child 1: age: \|_\|_\| years; gender: M/F  Child 2: age: \|_\|_\| years; gender: M/F  Child 3: age: \|_\|_\| years; gender: M/F  Child 4: age: \|_\|_\| years; gender: M/F  Child 5: age: \|_\|_\| years; gender: M/F  Child 6: age: \|_\|_\| years; gender: M/F  Child 7: age: \|_\|_\| years; gender: M/F  Child 8: age: \|_\|_\| years; gender: M/F  Child 9: age: \|_\|_\| years; gender: M/F  Child 10: age: \|_\|_\| years; gender: M/F |
| - 1. Please indicate the gender of the child about you are answering this questionnaire | Male  Female |
| - 1. Please indicate the age of the child you are answering this questionnaire | \|_\|_\| years |
| - 1. Referring to the child you are answering this questionnaire, is he/she vaccinated against human papillomavirus (HPV) (Gardasil/Gardasil 9/Cervarix? | Yes  No  I’m not sure |
| - 1. Are you vaccinated against HPV (Gardasil/Gardasil 9/Cervarix? | Yes  No  I’m not sure |

***Knowledge of HPV***

Please answer these questions about your knowledge on human papillomavirus

| - 1. Have you ever heard about HPV? | Yes  No *(If “No”, answer directly question 2.3)* |
| --- | --- |
| - 1. Where does the information you have about HPV come from? *(please indicate all that apply)* | Pediatrician  Family doctor  Gynecologist  Urologist  Nurse  Pharmacist  Other health care professionals  School  Friends/relatives  Newspapers/ magazines  Radio/ TV  Internet/ social media  Others |
| - 1. In your opinion, how do you think HPV infection is transmitted? *(please indicate all that apply)* | Sexually transmission  Blood transfusion  Use of public toilets  During pregnancy (mother-to-child transmission)  I’m not sure |

*Please indicate the level of your agreement with the following statements:*

|  | I totally disagree | I disagree | I agree | I totally agree | I don´t know |
| --- | --- | --- | --- | --- | --- |
| 2.4. HPV is the most common sexually transmitted disease |  |  |  |  |  |
| 2.5. HPV is a serious health problem |  |  |  |  |  |

| 2.6. In your opinion, who could get infected with HPV? *(please indicate all that apply)* | Girls  Women  Boys  Men  Nobody  I’m not sure |
| --- | --- |
| - 1. Which diseases do you think are related to HPV? *(please indicate all that apply)* | Bladder cancer  Genital warts  HIV/AIDS  Hepatitis  Infertility  Penis cancer  Irritable bowel syndrome  Cancer of the oral cavity  Esophagus cancer  Recurrent cystitis  Anal cancer  Vulvar cancer  Vaginal cancer  Cervical cancer  None of the above  I’m not sure |
| - 1. In your opinion, which measures can be taken to prevent transmission of HPV infection? *(please, indicate all that apply)* | Use of condoms  Vaccination against HPV  Late start of sexual relations  Good personal hygiene  Low sexual activity  Antibiotics  Birth control pill  It can’t be prevented  I don’t know |
| - 1. Who/where would you consult to obtain more information about HPV? *(please, indicate all that apply)* | Pediatrician  Family doctor  Gynecologist  Urologist  Nurse  Pharmacist  Other health care professionals  Teachers at school  Friends/relatives  Newspapers/ magazines  Radio/ TV  Internet/ social media  Others |

***General knowledge and acceptability of vaccines***

*Please indicate if you agree with the following statements:*

|  | I strongly disagree | I disagree | I agree | I strongly agree | I do not have enough information to answer |
| --- | --- | --- | --- | --- | --- |
| - 1. Vaccination is an effective measure to prevent infectious diseases in children and adults |  |  |  |  |  |
| - 1. Benefits thanks to vaccination are greater than risks |  |  |  |  |  |
| - 1. Vaccination is a useless measure |  |  |  |  |  |
| - 1. Parents not vaccinating their children put other people at risk |  |  |  |  |  |
| - 1. I’m afraid of vaccinating my children |  |  |  |  |  |

***Knowledge of HPV vaccine***

In section number 2 you have answered to items related to human papillomavirus knowledge. Please, answer now questions related to human papillomavirus vaccine

| - 1. Have you ever heard about HPV vaccine? | Yes  No |
| --- | --- |
| - 1. ¿ Where does your information about HPV vaccine come from? *(please, indicate all that apply)* | Pediatrician  Family doctor  Gynecologist  Urologist  Nurse  Pharmacist  Other health care professionals  Your child’s school  Friends/relatives  Newspapers/ magazines  Radio/ TV  Internet/ social media  Others |
| - 1. Is HPV vaccination included in the immunization calendar of your Autonomous Community? | Yes, only for girls  Yes, for boys and girls  It is not included  I’m not sure |
| - 1. What is the recommended age for HPV vaccine? | _ _ years  I’m not sure /a |
| - 1. In your opinion, who can receive HPV vaccine? *(please, indicate all that apply)* | Girls  Women  Boys  Men  Nobody  I’m not sure |

*Please indicate the level of agreement with the following statements:*

|  | I strongly disagree | I disagree | I agree | I strongly agree | I do not have enough information to answer |
| --- | --- | --- | --- | --- | --- |
| 4.6. HPV vaccine is effective |  |  |  |  |  |
| 4.7. The benefits of the HPV vaccine outweigh the risks |  |  |  |  |  |

| - 1. In your opinion, HPV vaccine prevents *(indicate all that apply)*: | Bladder cancer  Genital warts  HIV/AIDS  Hepatitis  Infertility  Penis cancer  Irritable bowel syndrome  Cancer of the oral cavity  Esophagus cancer  Recurrent cystitis  Anal cancer  Vulvar cancer  Vaginal cancer  Cervical cancer  None of the above  I’m not sure |
| --- | --- |

***Acceptability of HPV vaccine***

*Please indicate if you agree with the following statements:*

|  | I strongly disagree | I disagree | I agree | I strongly agree | I do not have enough information to answer |
| --- | --- | --- | --- | --- | --- |
| 5.1. HPV vaccination in girls is necessary |  |  |  |  |  |
| 5.2. HPV vaccine in boys is necessary |  |  |  |  |  |
| 5.3. The doctor recommends the HPV vaccine to me |  |  |  |  |  |
| *[Only if “No” or “I’m not sure” in 1.14*]  5.4. I would vaccinate my son/daughter against HPV |  |  |  |  |  |

| - 1. In case your answer to question 5.3 was “agree” or “fully agree”, could you please indicate which are the main reasons why you would vaccinate your son/daughter against HPV? *(please, indicate all that apply)* | To protect them against sexually transmitted diseases  To protect them against genital cancer and/or genital warts  To protect their future sexual partners against genital cancer and/or genital warts  I am aware of cancer and its prevention, because I know some cases amongst my closest relatives and friends.  To comply with the immunization schedule  It was the Doctor’s recommendation |
| --- | --- |
| - 1. In case your answer to question 5.3 was “disagree” or “fully disagree”, could you please indicate which are the main reasons why you would hesitate/refuse to vaccinate your son/daughter against HPV? *(please, indicate all that apply)* | I’m afraid of possible adverse effects  In my opinion, too many vaccines are given  My son/daughter is too young to be vaccinated  It is too late, my son/daughter has already had his/her first sexual intercourse  I don´t consider my son/daughter at risk of being infected with HPV  I prefer that my son/daughter decides later by him/herself to get vaccinated  My doctor told me vaccination is not necessary  I don’t have enough information about HPV vaccine  Vaccine price  I’d rather wait before taking this decision  There isn’t enough information about HPV vaccination yet  Other |
| - 1. In case your answer to question 5.3 was “disagree” or “fully disagree”, what type of information would you need to decide on vaccinating your son/daughter against HPV? *(please, indicate all that apply)* | Specific information about vaccine safety  Specific information about vaccine efficacy  General information about HPV  General information about HPV vaccine  Recommendation from my Doctor  Other |
| - 1. Who/where would you consult to obtain more information about HPV vaccine? *(please, indicate all that apply)* | Pediatrician  Family doctor  Gynecologist  Urologist  Nurse  Pharmacist  Other health care professionals  Your child’s school  Friends/relatives  Newspapers/ magazines  Radio/ TV  Internet/ social media  Others |

**Supplementary material 2****: Scores Keys assigned to each item to compute global scores**

- **HPV knowledge:**

**
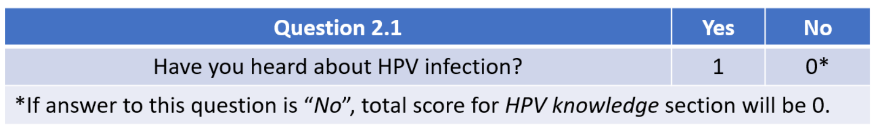
**


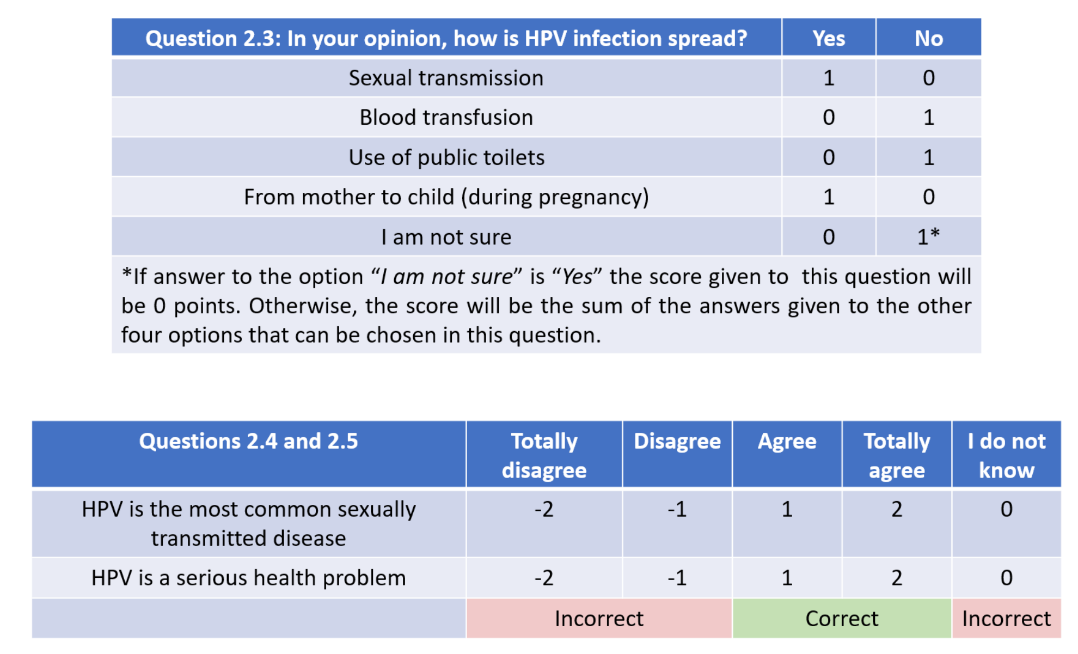


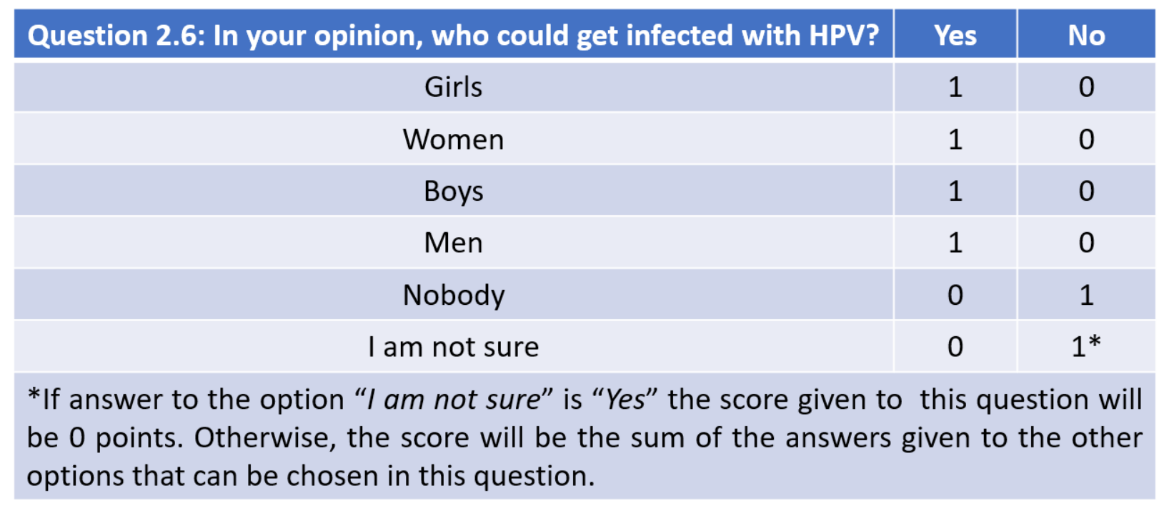


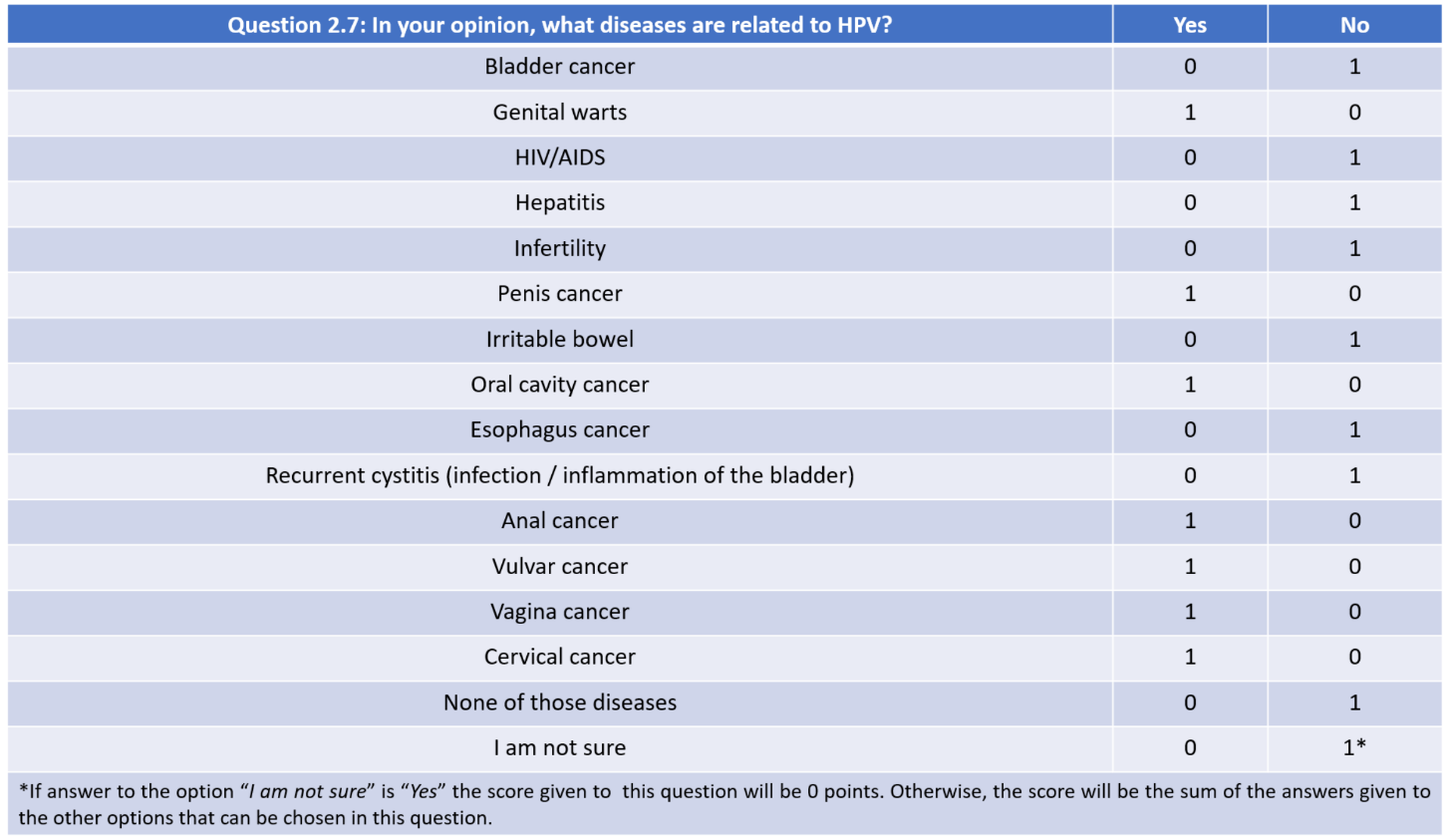


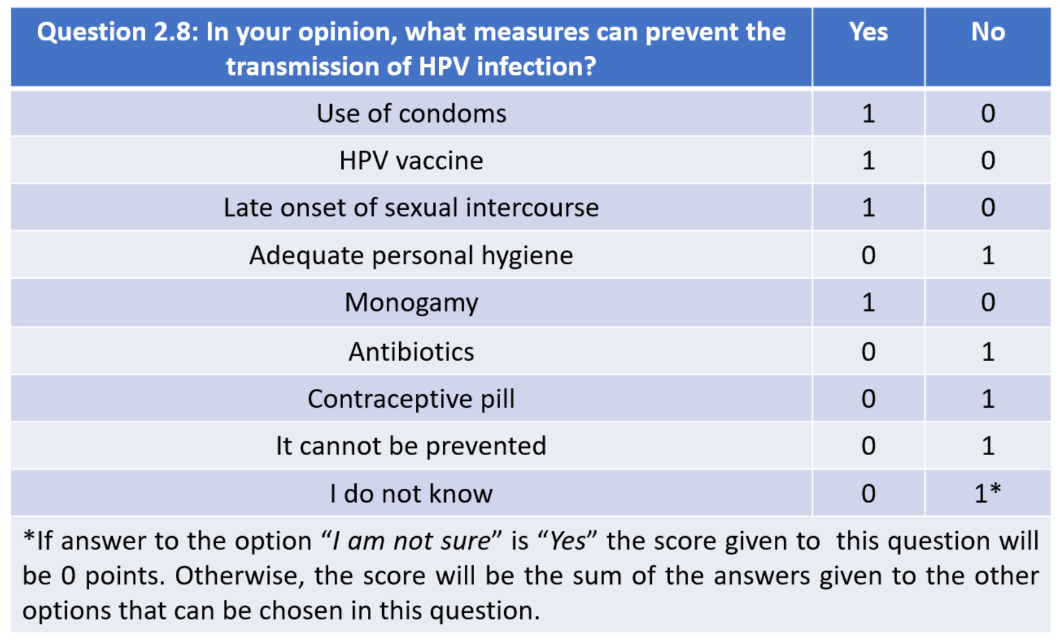


- **Vaccines acceptability and knowledge:**


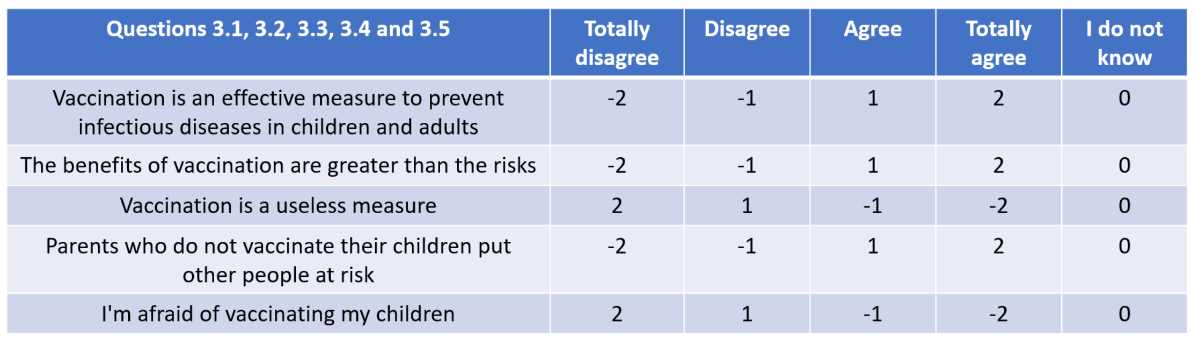


- **HPV vaccine knowledge:**

**
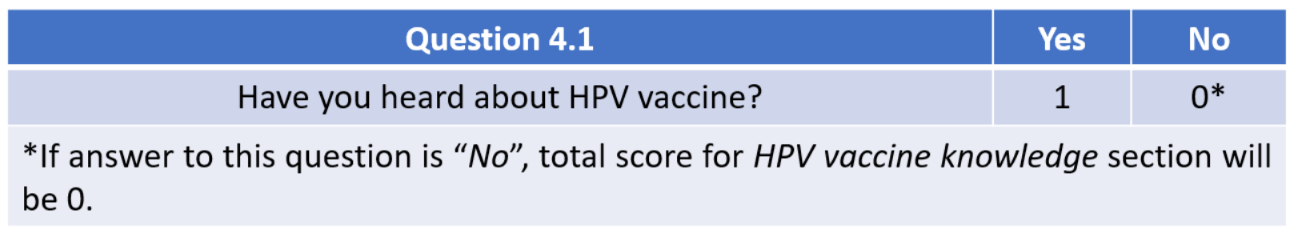
**


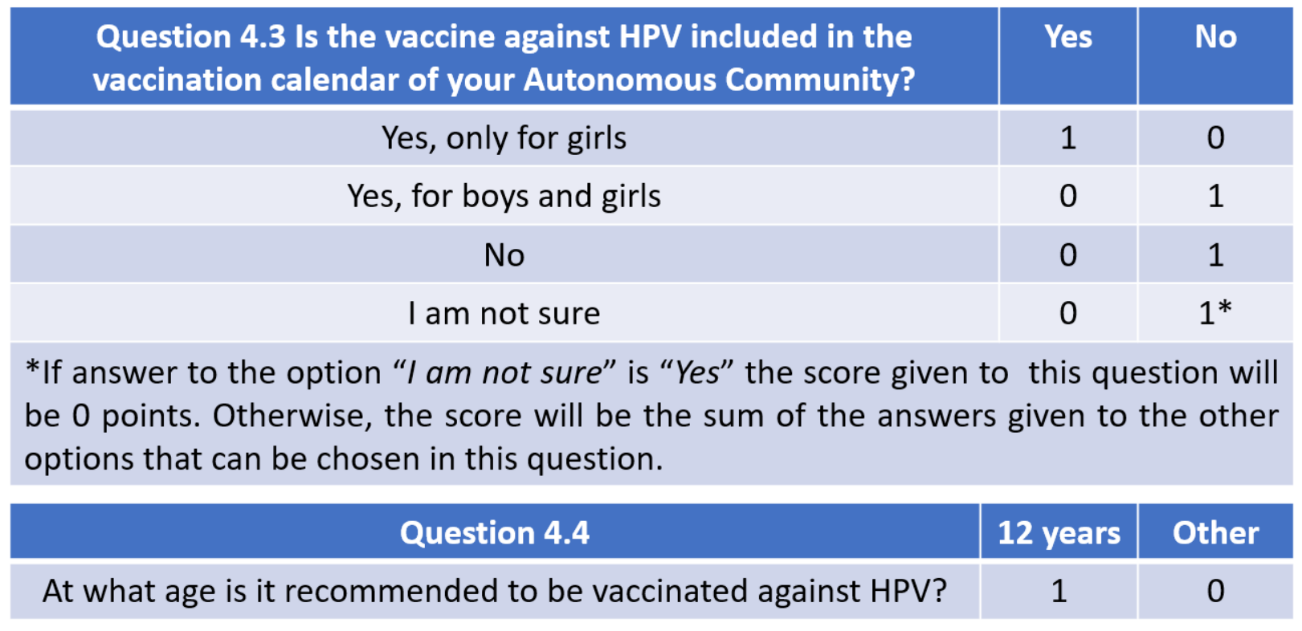


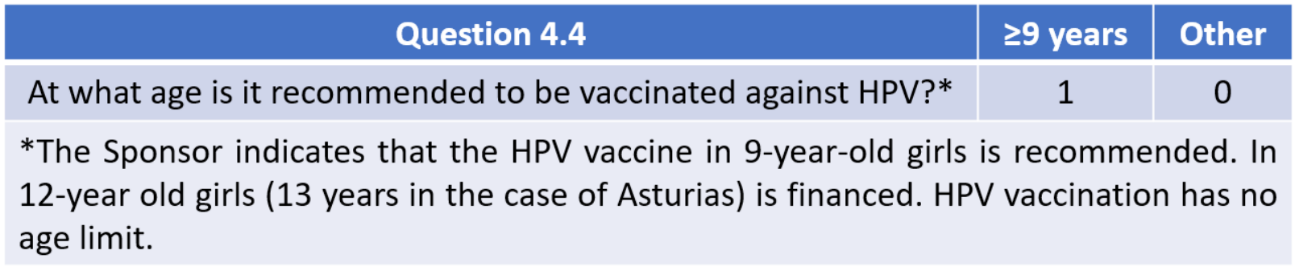


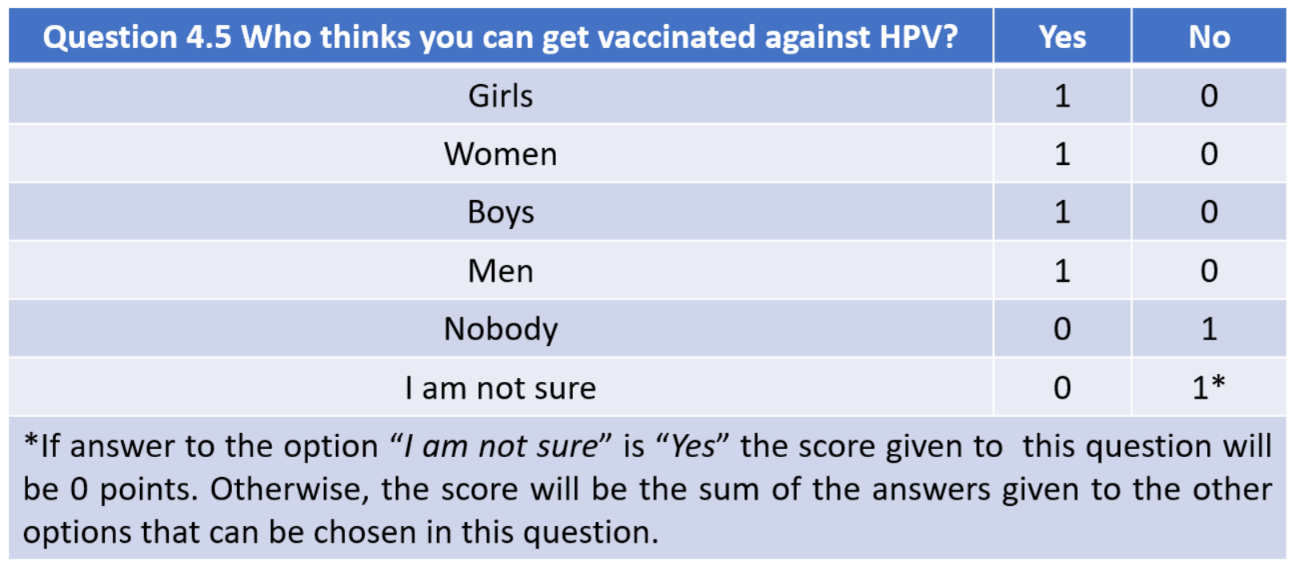


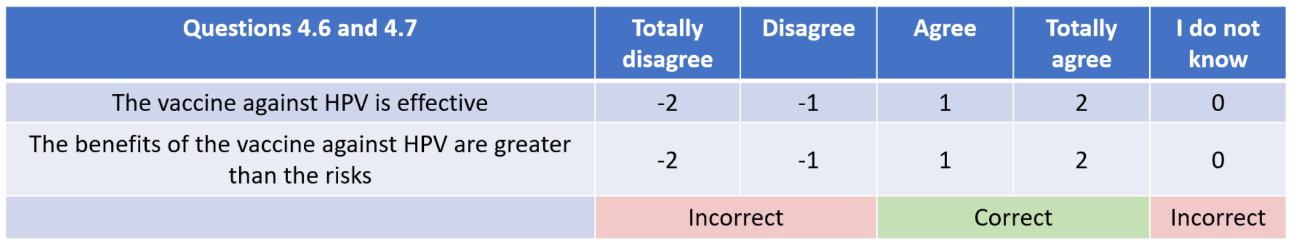


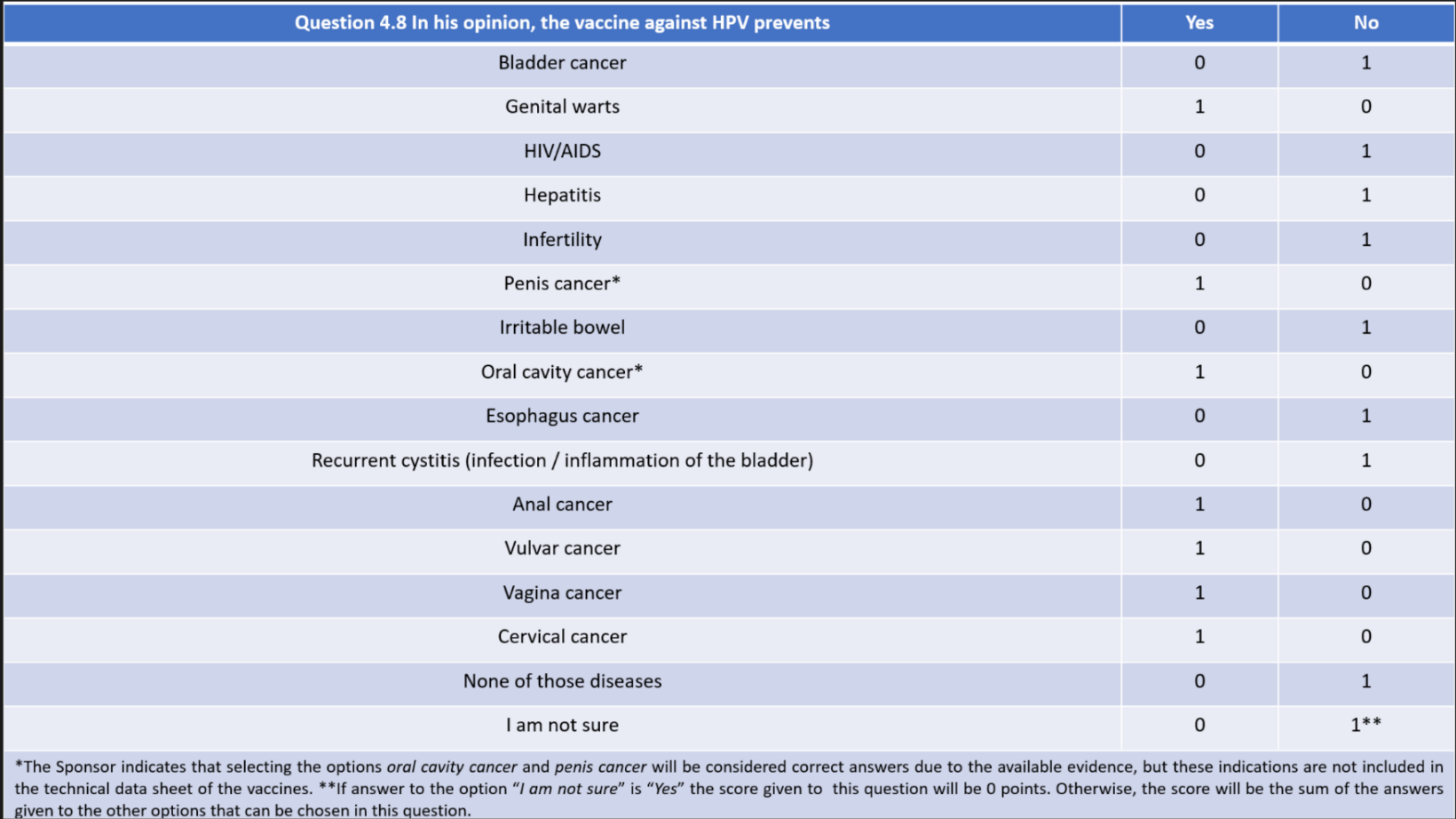


- **Acceptability of the HPV vaccine*:**

**
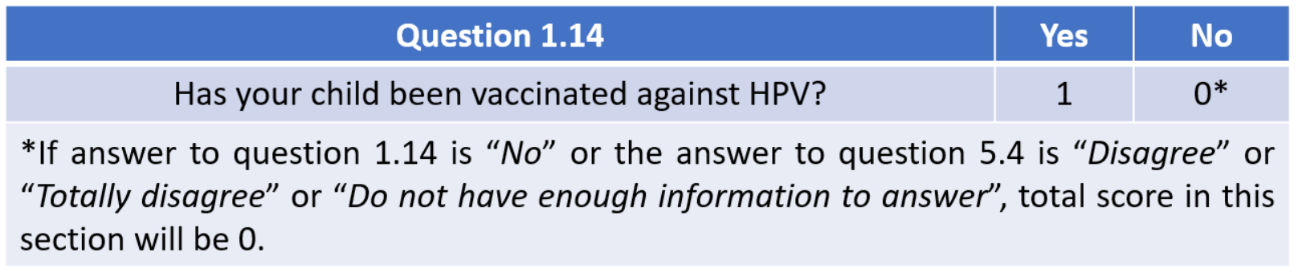
**


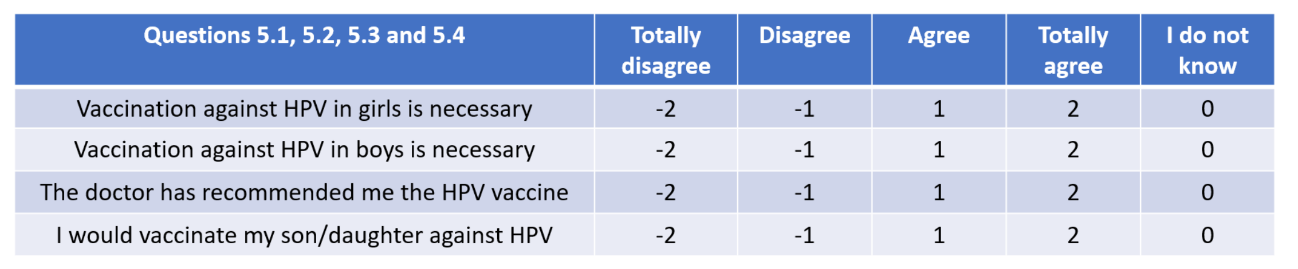


- **TOTAL**


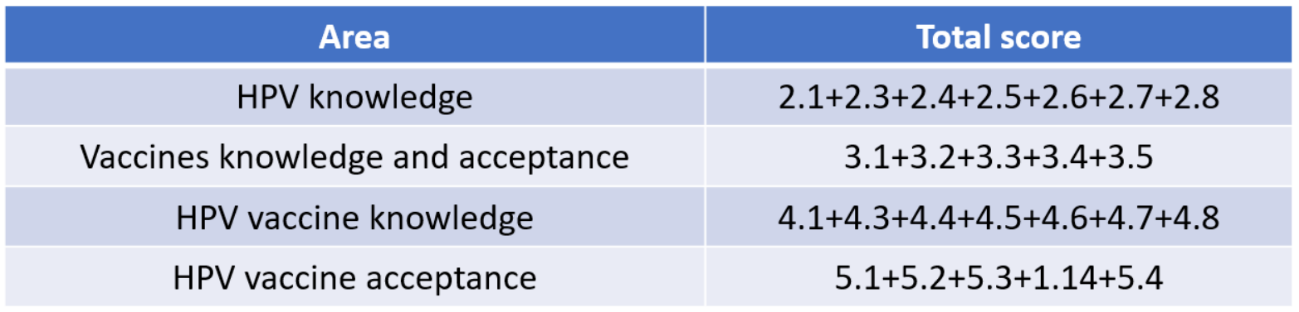


**Supplementary Material 3. Additional information of methodology and results**

**FIGURE S 1. Recruitment and data collection process.**

***
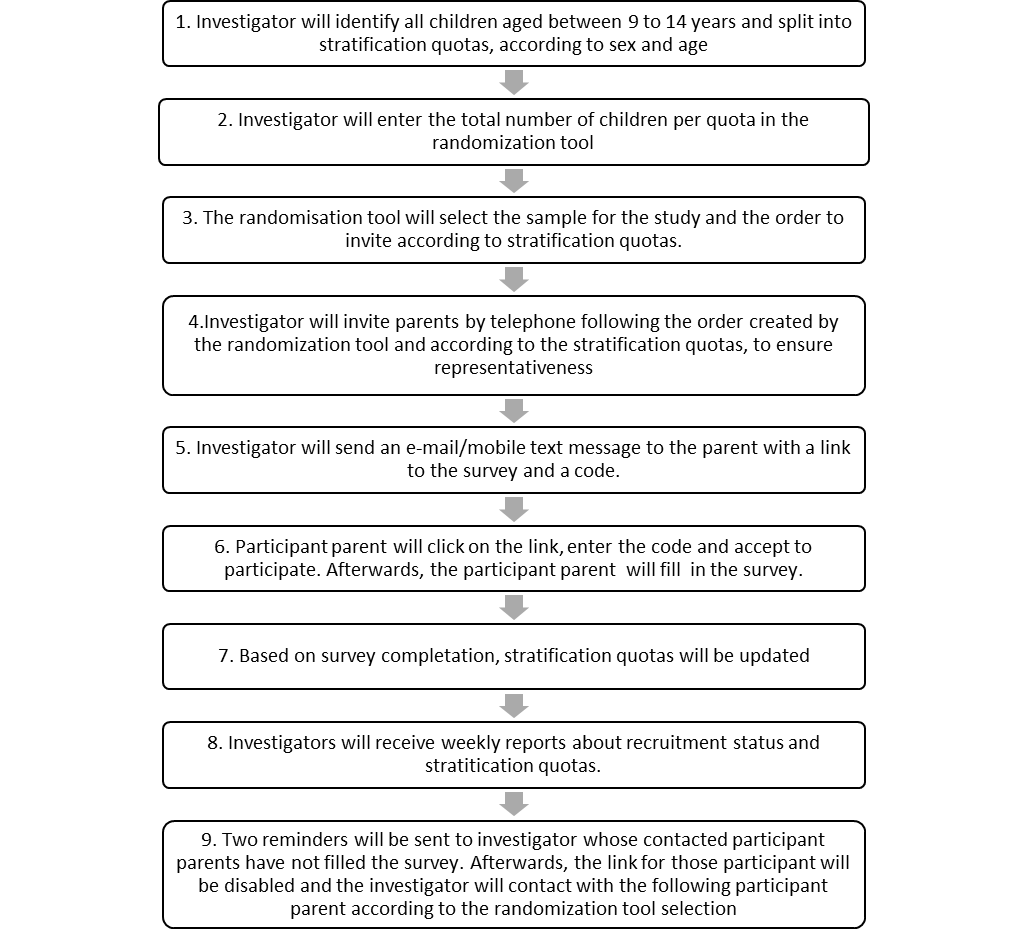
***

NOTE: For the paper-pen option, the participant could either collect the questionnaire in the office or print it directly from the same link. After completion, the questionnaire could be sent through pre-paid envelopes or be delivered at the doctor’s office. Per protocol, the survey could not be completed in the presence of the pediatrician, nor could anyone from his team interfere with the participant’s answers.

**FIGURE S 2.** Flowchart of participants.

| Selected (n=3110)  **Recruitment**  Excluded (n=1705):   - Unanswered (n=1071) - Refuse participation (n=555) - Surveys completed after 16-04-2020 (n=24) - Screening failure (n=5) - Other reasons (n=50)   **Surveyed**   - Participants with valid answers (n=1405):   - Online surveys (n=1116)   - Paper-and-pen surveys (n=289)     **Analyzed**   - Analyzed (n=1405) - Excluded (n=0) |
| --- |
| Note:   - *Unanswered (n=1071)*: 14 days have passed without completing the survey. - *Refuse participation* (n=555) - *Screening failure* (n=5): Surveys with participation code assigned, but later did not meet inclusion / exclusion criteria: - Did not meet criterion 1. Be the biological father, mother or legal tutor of at least one child (girl and/or boy) aged 9 to 14 years old (n=4). - Did not meet criterion 3. Be able to read, write and answer a questionnaire in Spanish (n=1). - *Other reasons* to be excluded (n=50):   - It cannot be guaranteed of which sibling in the family unit the survey was answered (n=15).   - Quality problems (n=18) (i.e., use of Tippex, surveys completed using a pencil, and parents contact procedure did not follow the necessary quality standards)   - High number of missing values (n=4).   - By mistake of the researcher, this survey was marked as completed when it was not actually completed (n=9).   - Both parents completed the survey (n=1)   - Technical issues (n=3). |

**FIGURE S 3. Total scores and distribution of respondents with regard to A) HPV knowledge, B) HPV vaccine knowledge, C) HPV vaccine acceptability, D) knowledge and acceptability of vaccines in general.**

| A. Total scores for the degree of knowledge about HPV infection. | | | | | | | | | | |
| --- | --- | --- | --- | --- | --- | --- | --- | --- | --- | --- |
| N | **Mean** | **95% CI Mean** | | **Med** | **SD** | **IQR** | **Skew** | **Kurt** | **Min** | **Max** |
|  |  | **Lower** | **Upper** |  |  |  |  |  |  |  |
| 1403 | 28.92 | 28.70 | 29.20 | 29.00 | 4.49 | 5.00 | -0.82 | 0.67 | 15.00 | 39.00 |
| 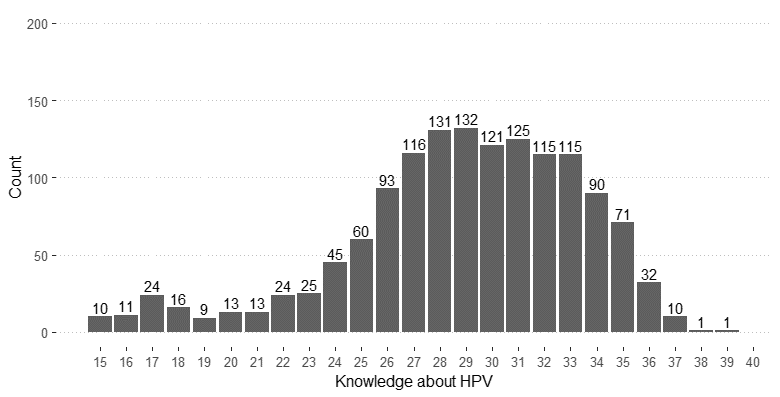 | | | | | | | | | | |

| B. Total scores for the degree of knowledge about the HPV vaccine. | | | | | | | | | | | |
| --- | --- | --- | --- | --- | --- | --- | --- | --- | --- | --- | --- |
| N | **Mean** | **95% CI Mean** | | **Med** | **SD** | **IQR** | **Skew** | **Kurt** | **Min** | **Max** |  |
|  |  | **Lower** | **Upper** |  |  |  |  |  |  |  |  |
| 1399 | 15.49 | 15.30 | 15.60 | 16.00 | 2.95 | 5.00 | -0.46 | -0.34 | 8.00 | 21.00 |  |
| 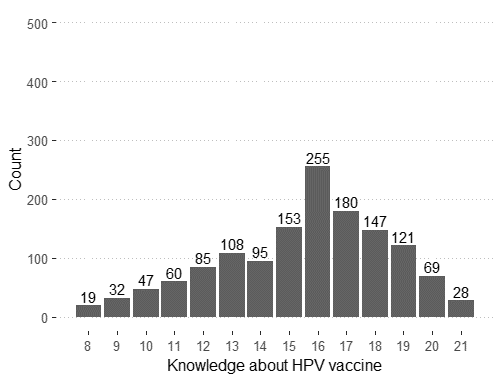 | | | | | | | | | | | |

| C. Total scores for the HPV vaccine acceptability. | | | | | | | | | | | |
| --- | --- | --- | --- | --- | --- | --- | --- | --- | --- | --- | --- |
| **N** | **Mean** | **95% CI Mean** | | **Med** | **SD** | **IQR** | **Skew** | **Kurt** | **Min** | **Max** |  |
|  |  | **Lower** | **Upper** |  |  |  |  |  |  |  |  |
| 1398 | 3.37 | 3.30 | 3.44 | 4.00 | 1.36 | 1.00 | -0.90 | 0.16 | 0.00 | 5.00 |  |
| 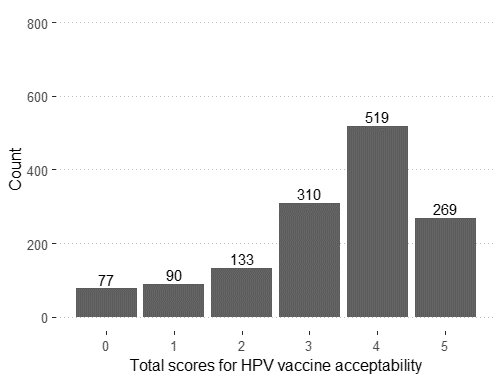 | | | | | | | | | | | |

| D. Total scores for the degree of knowledge and acceptance of the vaccines in general. | | | | | | | | | | | |
| --- | --- | --- | --- | --- | --- | --- | --- | --- | --- | --- | --- |
| N | **Mean** | **95% CI Mean** | | **Med** | **SD** | **IQR** | **Skew** | **Kurt** | **Min** | **Max** |  |
|  |  | **Lower** | **Upper** |  |  |  |  |  |  |  |  |
| 1402 | 6.61 | 6.44 | 6.78 | 7.00 | 3.19 | 5.00 | -0.84 | 0.27 | -6.00 | 10.00 |  |
| 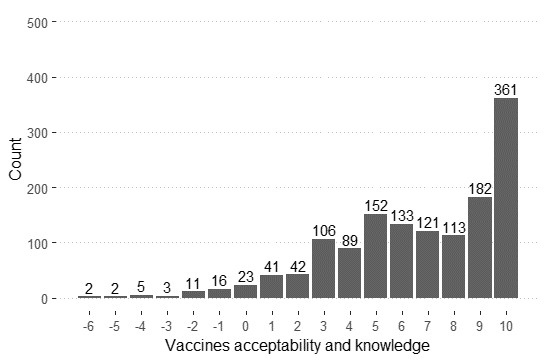 | | | | | | | | | | | |

Histogram representation. Scores ranges: HPV knowledge (0 to 40), knowledge and acceptance of vaccines in general (-10 to 10), HPV vaccine knowledge (0 to 21) and HPV vaccine acceptability (0 to 5). Counts represent respondents. CI: Confidence Interval. SD: Standard deviation. Med: Median. IQR: Interquartile range. Skew: Skewness. Kurt: Kurtosis. Missing data were not imputed (listwise method).

**FIGURE S 4. Boxplot representations of total scores.**

A) degree of Human Papillomavirus (HPV) knowledge about HPV infection, B) degree of knowledge about HPV vaccination, C) degree of acceptability of the HPV vaccine, D) degree of knowledge and acceptability of vaccines in general. Boxplot representations of scores with regard to HPV knowledge (score range: 0 to 40), knowledge and acceptability of vaccines in general (score range: -10 to 10), HPV vaccine knowledge (score range: (0 to 21) and HPV vaccine acceptability (score range: 0 to 5). Line inside the box represents the median. Lower and upper boundaries of each box indicate Q1 and Q3, respectively.

***
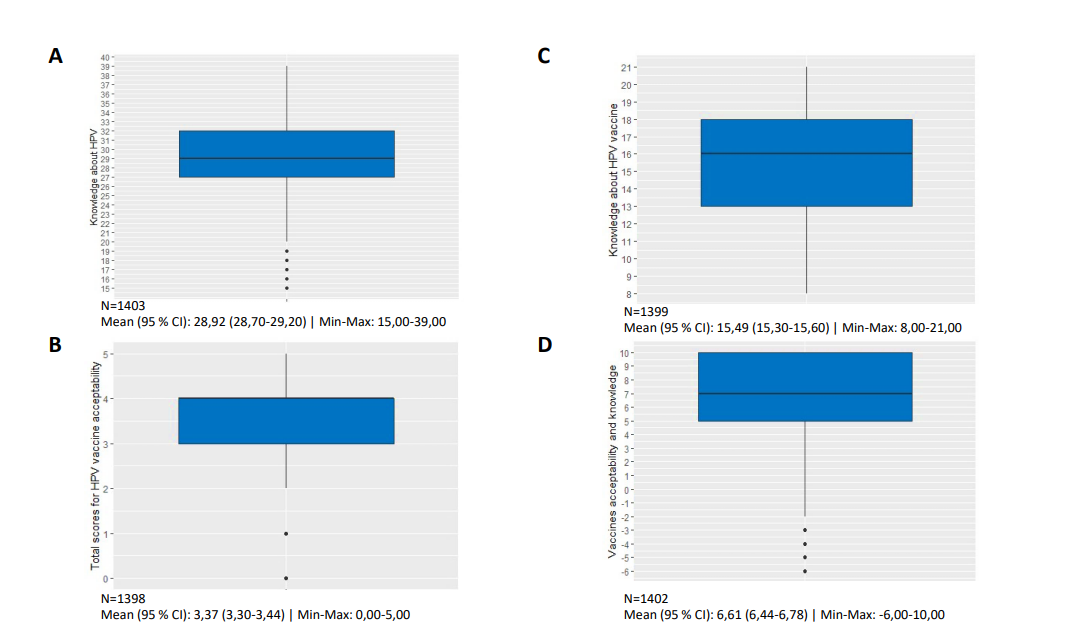
***

**FIGURE S 5. Wrong/Right answers for HPV knowledge (section 2).**

The participants' answers were analyzed and classified according to whether they were correct or incorrect. *Agree/disagree questions.

**FIGURE S 6.** Wrong/Right answers for HPV vaccine knowledge (section 4).

The participants' answers were analyzed and classified according to whether they were correct or incorrect. *Agree/disagree questions.

**Figure S 7: Reasons to vaccinated and to not vaccinate the child.**


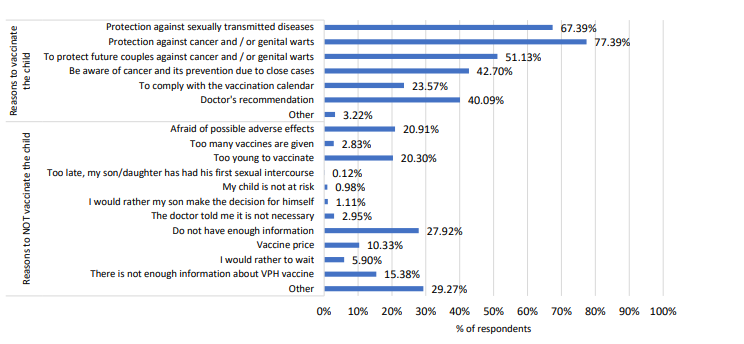


**Figure S 8**: **Responses to questions related to HPV vaccine acceptability**


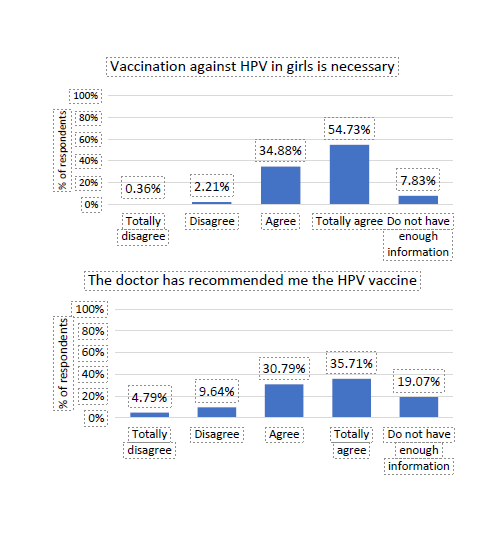

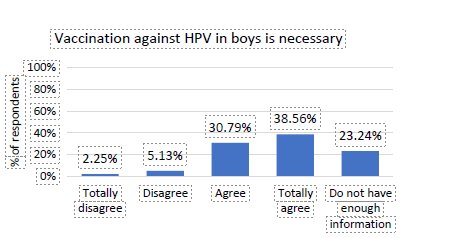


**Figure S 9: Responses to questions related to knowledge and acceptability of vaccines in general**


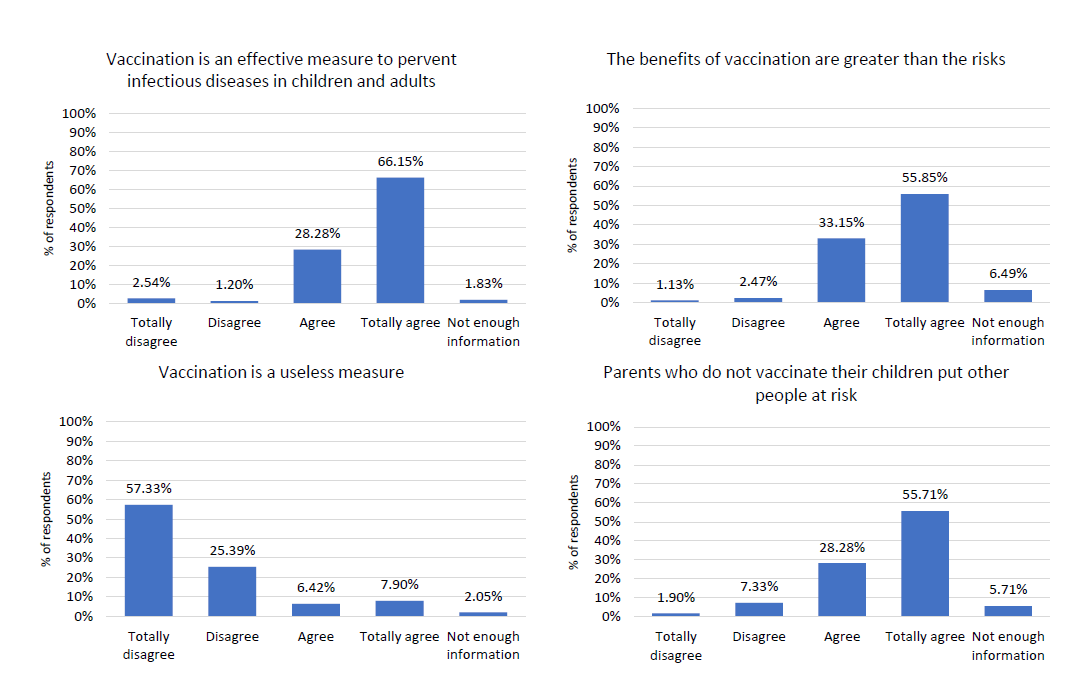

Supplement: Supplementary file 1 — Additional file 1: Material S1. Questionnaire used in the KAPPAS study. Material S2. Scores Keys assigned to each item to compute global scores. Material S3. Additional information of methodology and results. Figure S1. Recruitment and data collection process. Figure S2. Flowchart of participants. Figure S3. Total scores and distribution of respondents with regard to A) HPV knowledge, B) HPV vaccine knowledge, C) HPV vaccine acceptability, D) knowledge and acceptability of vaccines in general. Figure S4. Box representation of total scores. Figure S5. Wrong/Right answers for HPV knowledge. Figure S6. Wrong/Right answers for HPV vaccine knowledge. Figure S7. Reasons to vaccinated and to not vaccinate the child. Figure S8: Responses to questions related to HPV vaccine acceptability. Figure S9: Responses to questions related to Knowledge and acceptability of vaccines in general. [file 13027_2022_467_MOESM1_ESM.docx]
